# What keeps medical students healthy and well? A systematic review of observational studies on protective factors for health and well-being during medical education

**DOI:** 10.1186/s12909-019-1532-z

**Published:** 2019-04-01

**Authors:** Thomas Kötter, Stephan Fuchs, Marcus Heise, Henna Riemenschneider, Linda Sanftenberg, Christian Vajda, Karen Voigt

**Affiliations:** 1 Institute of Social Medicine and Epidemiology, University of Lübeck, Lübeck, Germany; 2 Institute of General Practice & Family Medicine, Martin-Luther University Halle-Wittenberg, Halle, Germany; 3 Department of General Practice, Carl Gustav Carus Faculty of Medicine, Dresden, Germany; 4 Institute of General Practice and Family Medicine, University Hospital, Ludwig-Maximilians-University (LMU), Munich, Germany; 5 Department of Medical Psychology and Psychotherapy, Medical University of Graz, Graz, Austria

**Keywords:** Schools, medical, Students, medical, Education, medical, Health promotion, Longitudinal studies

## Abstract

**Background:**

Despite the growing evidence of a negative impact of medical school on students’ health and well-being, little is known about protective factors for staying healthy and well during medical education. Therefore, a systematic review of peer-reviewed studies aiming to identify such predictors was conducted.

**Methods:**

Medline, Embase, and PsychInfo were systematically searched by using preselected MeSH terms to identify English- and German-language peer-reviewed articles (observational studies) examining predictors for medical students’ health and well-being, published between January 2001 and April 2018. Two authors independently selected abstracts reporting predictors for medical students’ health and well-being. Further, two authors extracted information from the identified studies, needed for methodological quality assessment of the studies, as well as for comprehensive description of identified predictors.

**Results:**

From 5013 hits in the database search, six observational studies met the inclusion criteria and were included in the final analysis. These studies were of heterogeneous design and quality. They featured a wide variety of health and well-being related outcomes and of its predictors. Lower levels of perceived stress, as well as lower levels of neuroticism were found to predict better health-related outcomes.

**Conclusions:**

Further research, by using harmonized tools for the assessment of outcomes, as well as predictors, is needed to determine what keeps students healthy and well during medical education. Identifying protective factors is an essential prerequisite for the design of evidence-based health-promoting interventions.

**Electronic supplementary material:**

The online version of this article (10.1186/s12909-019-1532-z) contains supplementary material, which is available to authorized users.

## Background

Studying medicine can pose a threat to students’ health and well-being. A perceived highly competitive environment, the challenges imposed by confrontations with disease, suffering, and death, a high workload, and a lack of social support are among the factors that contribute to a declining general and mental health during medical education. Certain personality traits, above all neuroticism, and coping styles (e.g., emotion-focused coping) seem to be among further pathogenetic factors [1–3].

Apart from the personal suffering, physicians’ impaired health and well-being may have a negative impact on the quality and availability of health services [4]. Against this background, the importance of physician self-care has been included in the most recent version of the Declaration of Geneva [5].

Effective interventions are needed to address the decrease of health and well-being among medical students during medical school. To design and implement evidence-based health-promoting interventions, we need to know what influenceable factors keep medical students healthy and well during their studies.

To date, a conclusive summary of protective factors for health and well-being during medical education is lacking.

### Objective

Therefore, we conducted a systematic review of peer-reviewed studies with the aim of identifying such factors, that is, predictors of staying healthy and well during medical school.

## Methods

### Protocol and registration

We used a standard review protocol, which was submitted to the funding body prior to the start of the study.

### Eligibility criteria

We aimed to summarise studies identifying predictors for health -related outcomes in the natural course. For this, longitudinal, observational studies are appropriate [6]. Therefore, longitudinal, observational studies published in English or German that reported any protective factors for medical students’ health and well-being were included, regardless of length of follow-up and using any outcomes related to health and well-being of medical students. We excluded cross-sectional studies, interventional studies, reviews, qualitative research, studies not targeting medical students, and publications not reporting results. Medical curricula, admission criteria and the context of medical education change over time. Taking into account the respective impact on predictors for health and well-being, we restricted the search to articles published since January 2001.

### Information sources

Electronic databases, including MEDLINE, EMBASE, and PsychInfo, were searched, through the Ovid platform [7] (last update: July 2018).

### Search

Items for the search algorithm (Additional file 1) were collected and collated in a multifold expert group procedure based on existing MeSH terms. We screened the reference lists of included studies and previous systematic reviews on similar topics.

### Study selection

Two review authors (HR and KV) independently evaluated all titles and abstracts for eligibility (cf. Eligibility criteria). We resolved disagreements by consensus or discussion with a third reviewer (CV). If multiple reports described the same study, we chose the most recent full-text publication in a peer-reviewed journal as the main report. Two authors (SF and MH) screened these selected full texts for eligibility (cf. Eligibility criteria) with disagreements being resolved by a third reviewer (LS).

### Data collection process

Two authors (TK and SF) independently extracted data from the full-text articles. They used a self-developed, piloted extraction form accompanied by a codebook created for this review. Reviewers resolved disagreements by consensus or through discussion with a third author (MH).

### Data items

We extracted data regarding study sites, duration, outcomes and independent variables, number of participants, response rate and lost-to-follow-up, statistical methods and risk estimates, conclusions and limitations of the included studies.

### Risk of bias in individual studies

Two authors (TK and SF) independently assessed the quality of the included reports by using the Newcastle-Ottawa Scale [8]. Disagreements were resolved by discussion with a third reviewer (MH) and subsequent consensus.

## Results

For the PRISMA (Preferred Reporting Items for Systematic Reviews and Meta-Analyses) checklist for this report, see Additional file 2 [9].

### Study selection

**Fig. 1:**
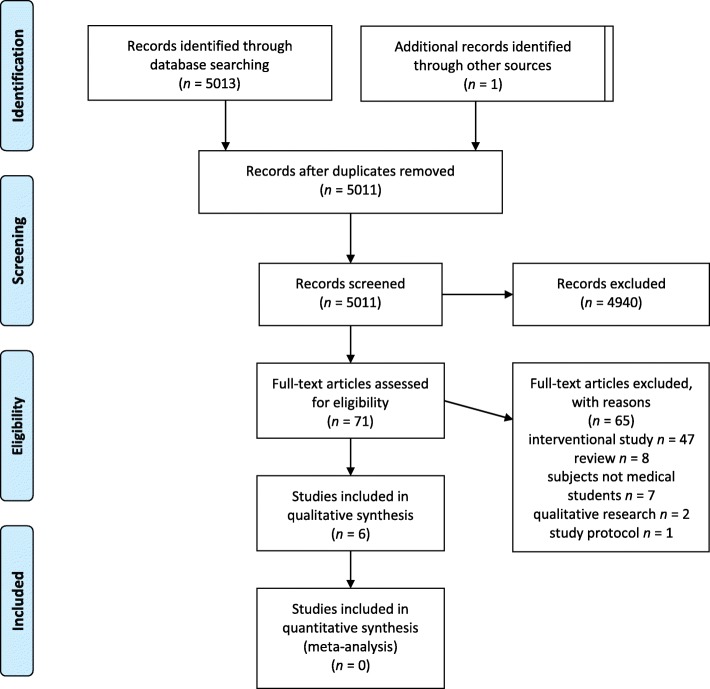
PRISMA flowchart

We identified 5013 references in our literature search, of which 71 studies were potentially eligible for the systematic review. Of these, 65 studies did not meet the inclusion criteria. Six reports [1, 10–14] were included in the full-text analyses (see PRISMA flowchart [Fig. 1]).

### Study characteristics

The identified six studies were conducted in the United States (US; 2), Germany (2), Norway (1), and Malaysia (1). All studies were published as full-text English-language peer-reviewed journal articles between 2006 and 2016. The median duration of observation was one year (range: 0.75 to 6). The size of the longitudinal sample ranged from 42 to 792 (median: 229.5).

### Risk of bias within studies

The methodological quality of the included studies was heterogeneous and overall mediocre (see Additional file 3: Table S1). The assessment of potential predictors and outcomes was based on students’ self-written reports in all studies. No study employed objective measurements of health and well-being. Two studies had a follow-up rate < 50% and did not describe those lost [10, 11], one study did not report the follow-up rate [12]. There is no common minimum length of follow-up for studies aiming to identify predictors for health-related outcomes. But even assuming a relatively short period of follow-up of one year, two studies failed to reach this period [13, 14].

### Results of individual studies

The studies featured a wide variety of outcomes and potential predictors (see results of individual studies in Additional file 4: Table S2). One study ([1]) used life satisfaction and another study ([13]) chose general health as single outcomes. All other studies employed outcomes more or less closely related to mental health (resilience [i.e., absence of burnout [10]], anxiety and depression [11–13], emotional, personal, and social well-being [14], stress [12]). One tool was used in more than one study: the Hospital Anxiety and Depression Scale (HADS) [15]. Other tools used for outcome assessment were the Maslach Burnout Inventory (MBI) [16], the short-form version of the Depression Anxiety Stress Scale (DASS-21) [17], and the short form of the Mental Health Continuum (MHC-SF) [18].

Amongst potential predictors for the aforementioned outcomes, personal characteristics/personality, perceived (medical school) stress/stressors, coping, social support, and sociodemographic variables were assessed. The authors of the included studies used an even wider variety of tools and questions for the assessment of potential predictors (see in Additional file 4: Table S2).

This diversity of the employed questionnaires led to few consistent results across the studies. Lower levels of neuroticism were identified as protective against the decline of health and well-being during medical education in two studies [12, 13]. Lower levels of subjective medical school-associated stress were correlated with better health outcomes in two studies [1, 11]. Among other factors connected to health/well-being at the end of the included longitudinal studies, were adequate coping (e.g., less emotion-focused coping [1], regular physical activity [13]), sociodemographic characteristics (lower age [13], being non-white [10]), and factors closely related to medical education itself (increased satisfaction with the learning environment [10], greater agreement that student education is a priority for faculty members [10], lower striving for perfection [13], better ability to distance oneself from medicine [13], no overexertion [11]).

The effects were overall small to moderate.

### Synthesis of results

Due to the heterogeneity of potential predictors and outcomes, it was not feasible to conduct any meta-analyses.

## Discussion

### Summary of evidence

In our systematic review of observational studies that aimed to identify modifiable predictors for good health and/or well-being of medical students, we found few consistent results. The included studies concur in identifying protective factors such as low neuroticism, perceived low medical school-associated stress, and sustainable coping strategies.

The two included observational studies with the longest follow-up period found subjective general and medical school-associated stress to be a predictor for students’ general and mental health [1, 11]. In the light of universally accepted stress theories, this is a plausible result [19]. Furthermore, it constitutes a starting point for health-promoting activities. Stress reduction and stress management interventions for medical students have been extensively studied [20] and found to be effective for health and well-being related outcomes. However, most of the identified observational studies focused on the individual medical student and literature on setting-based interventions are scarce.

The results of the included studies imply that a positive attitude of teaching and administrative staff towards medical students could promote and protect the students’ health and well-being [1, 10], as also stated by Slavin [21]. As it is likely that an important prerequisite of such a positive attitude is the good health and well-being of the staff itself, setting-based interventions and health-promotion strategies should aim at all status groups in medical schools.

Low neuroticism has been shown to be a strong protective factor for health and well-being in many other contexts [22–24]. As personality traits are relatively stable by the age most students enter medical school, “treating” neuroticism is a less promising health-promoting intervention when compared to conveying stress management skills. Mediating effects in regard to neuroticism and subjective well-being could be reached through anti-stress techniques, for example, mindfulness training [25, 26].

### Strengths and limitations

To our knowledge, this is the first systematic review of protective factors for medical students’ health and well-being. This systematic review has been conducted by following a rigorous methodological approach. As our review includes studies from different geographical and cultural contexts (US, Europe, Malaysia), it needs to be considered that the perception of health and well-being can vary in different cultures.

Except for two studies [1, 11], none of the included studies reported an observation period longer than one year. This clearly limits the results with regard to the research question. As the studies showed a low level of comparability among each other, for example, regarding the employed variables and tools, we could not undertake a meta-analysis. The validity of our results is limited by the overall mediocre quality of the included studies. Although it has been shown earlier that self-reported health and well-being is associated with objective health indicators [27], the lack of the latter in the included studies may pose another limitation. This is particularly important, because medical students are socialized to neglect their own health problems during their education [28].

### Implications for research

Further research, by using harmonized tools for outcomes, as well as predictors, is needed to determine what keeps students healthy during medical education. Most of the studies not included in this review focused on short-term or one-time interventions (e.g., mentoring or coaching programs, self-awareness workshops, mind-body groups, etc.), which were evaluated shortly after their implementation. However, these studies do not make any statements about the long-term effects on students’ health and well-being. Furthermore, these studies relied solely on self-reported data, which are susceptible to a possible social desirability bias. Future studies should be designed to cover the whole period of studies from the beginning until graduation and should include behavioral measurements as outcomes when possible. Not only because of the large numbers of participants needed for such long-term observational studies but also because of the enhanced validity and the possibility of comparing different curriculums, studies should be designed to be multi-center studies.

### Implications for practice

Identifying protective factors is an essential prerequisite for the design of evidence-based health-promoting interventions. Based on the existing knowledge, stress management and coping seminars, as well as measures enhancing the attitude of all status groups in medical schools towards the importance of a high-quality education but also healthy learning environment seem to be promising health-promoting interventions [21]. To prevent a long-term negative impact on health services quality, these interventions should address the specific needs of students with high neuroticism and should ensure their well-being during and after medical school.

## Conclusions

Less neurotic persons, students with sustainable stress coping and good stress management skills, as well as healthy medical school environments that appreciate students and their educational performances increase the odds of staying healthy and well during the course of studies. This is of great value also for promoting a healthy work force in the future. More research is needed to be able to create an evidence-based model for medical students’ health and well-being, which could serve as a framework for the design and evaluation of health-promoting interventions for medical students.

## Additional files

Additional file 1: Search algorithm. (PDF 35 kb)

Additional file 2: PRISMA Checklist. (PDF 191 kb)

Additional file 3: **Table S1.** Results of the quality assessment. (PDF 20 kb)

Additional file 4: **Table S2.** Characteristics of the included studies. (PDF 44 kb)

## Abbreviations

DASS-21: Depression Anxiety Stress Scale
HADS: Hospital Anxiety and Depression Scale
MBI: Maslach Burnout Inventory
MHC-SF: Mental Health Continuum
PRISMA: Preferred Reporting Items for Systematic Reviews and Meta-Analyses

## References

[1] Kjeldstadli K, Tyssen R, Finset A, Hem E, Gude T, Grønvold NT (2006). Life satisfaction and resilience in medical school - a six-year longitudinal, nationwide and comparative study. BMC Med Educ.

[2] Waldmann C, Wolfradt U, Klement A, Fuchs S, Riemenschneider H, Heise M (2017). Zum Zusammenhang zwischen Persönlichkeitsmerkmalen und Burnout-Risiken. Prävent Gesundheitsförderung..

[3] Boni RADS, Paiva CE, de Oliveira MA, Lucchetti G, Fregnani JHTG, Paiva BSR (2018). Burnout among medical students during the first years of undergraduate school: prevalence and associated factors. PLoS One.

[4] Wallace JE, Lemaire JB, Ghali WA (2009). Physician wellness: a missing quality indicator. Lancet..

[5] WMA - The World Medical Association - Declaration of Geneva. https://bit.ly/2yosyvo. Accessed 18 Mar 2019.

[6] Gordis L (2014). Epidemiology.

[7] Ovid platform. http://www.ovid.com/. Accessed 18 Mar 2019.

[8] Wells G, Shea B, O’Connell D, Peterson J, Welch V, Losos M, et al. The Newcastle-Ottawa scale (NOS) for assessing the quality of nonrandomised studies in meta-analyses. https://bit.ly/1SkJ3w3. Accessed 18 Mar 2019.

[9] Moher D, Liberati A, Tetzlaff J, Altman DG (2009). The PRISMA group. Preferred reporting items for systematic reviews and meta-analyses: the PRISMA statement. PLoS Med.

[10] Dyrbye LN, Power DV, Massie FS, Eacker A, Harper W, Thomas MR (2010). Factors associated with resilience to and recovery from burnout: a prospective, multi-institutional study of US medical students. Med Educ.

[11] Voltmer E, Kötter T, Spahn C (2012). Perceived medical school stress and the development of behavior and experience patterns in German medical students. Med Teach.

[12] Yusoff MSB, Esa AR, Mat Pa MN, Mey SC, Aziz RA, Abdul Rahim AF (2013). A longitudinal study of relationships between previous academic achievement, emotional intelligence and personality traits with psychological health of medical students during stressful periods. Educ Health Abingdon Engl.

[13] Kötter T, Tautphäus Y, Obst KU, Voltmer E, Scherer M (2016). Health-promoting factors in the freshman year of medical school: a longitudinal study. Med Educ.

[14] Michalec B, Keyes CLM (2013). A multidimensional perspective of the mental health of preclinical medical students. Psychol Health Med.

[15] Zigmond AS, Snaith RP (1983). The hospital anxiety and depression scale. Acta Psychiatr Scand.

[16] Maslach C, Jackson SE (1986). The Maslach burnout inventory manual.

[17] Henry JD, Crawford JR (2005). The short-form version of the depression anxiety stress scales (DASS-21): construct validity and normative data in a large non-clinical sample. Br J Clin Psychol.

[18] Lamers SMA, Westerhof GJ, Bohlmeijer ET, ten Klooster PM, Keyes CLM (2011). Evaluating the psychometric properties of the mental health continuum-short form (MHC-SF). J Clin Psychol.

[19] Lazarus RS, Folkman S (1984). Stress, appraisal, and coping.

[20] Shiralkar MT, Harris TB, Eddins-Folensbee FF, Coverdale JH (2013). A systematic review of stress-management programs for medical students. Acad Psychiatry.

[21] Slavin SJ (2016). Medical student mental health: culture, environment, and the need for change. JAMA.

[22] Srivastava K, Das RC (2015). Personality and health: road to well-being. Ind Psychiatry J.

[23] Steel P, Schmidt J, Shultz J (2008). Refining the relationship between personality and subjective well-being. Psychol Bull.

[24] Gale CR, Booth T, Mõttus R, Kuh D, Deary IJ (2013). Neuroticism and extraversion in youth predict mental wellbeing and life satisfaction 40 years later. J Res Personal.

[25] Wenzel M, von Versen C, Hirschmüller S, Kubiak T (2015). Curb your neuroticism – mindfulness mediates the link between neuroticism and subjective well-being. Personal Individ Differ.

[26] de Vibe M, Solhaug I, Rosenvinge JH, Tyssen R, Hanley A, Garland E (2018). Six-year positive effects of a mindfulness-based intervention on mindfulness, coping and well-being in medical and psychology students; results from a randomized controlled trial. PLoS One.

[27] Schnittker J, Bacak V. The increasing predictive validity of self-rated health. PLoS One. 2014;9. 10.1371/journal.pone.0084933. 10.1371/journal.pone.0084933 PMC3899056 24465452

[28] Kötter T, Obst K, Voltmer E (2017). Präsentismus bei Medizinstudierenden. Prävent Gesundheitsförderung.

