# Supplementary material for: What keeps medical students healthy and well? A systematic review of observational studies on protective factors for health and well-being during medical education
Source: BMC Med Educ. 2019 Apr 1;19:94. doi: 10.1186/s12909-019-1532-z (PMC6444449; doi:10.1186/s12909-019-1532-z)
Supplement: Supplementary file 1 — Search algorithm. (PDF 35 kb) [file 12909_2019_1532_MOESM1_ESM.pdf]

Search algorithm for MEDLINE, EMBASE and PsycInfo via the Ovid platform

Search terms within the columns were combined using “OR”, the three colums were combined using “AND”.

protective factor*	burnout	medical student*
health promotion	psychological stress	medical school
salutogenesis	life satisfaction	medical education
prevention	depression	medical faculty
primary prevention	distress	medical studies
health behavior	anxiety	students, medical
social support	health status	schools, medical
coping	health related quality of life	education, medical
adaption, psychological	quality of life	
mental health	fitness	
life style	satisfaction	
resilience, psychological	fatigue	
financial support	sleep* quality	
health pomoting factor*	suicidality	
personality	mental disorder*	
wellbeing	exam nerves	
health promoting activit*	exam anxiety	
spirituality	drug abuse	
physical activit*	happiness	

role model	mental wellbeing	
mentoring	mindfulness	
health-promoting behavi*	health belief	
health literacy	self efficacy	
health education		
predictive factor*		
identification		
work life balance		
curriculum		
exam		
vaccination		
nutrition		
sti prevention		
immunization		
