# Supplementary material for: What keeps medical students healthy and well? A systematic review of observational studies on protective factors for health and well-being during medical education
Source: BMC Med Educ. 2019 Apr 1;19:94. doi: 10.1186/s12909-019-1532-z (PMC6444449; doi:10.1186/s12909-019-1532-z)
Supplement: Supplementary file 3 — Table S1. Results of the quality assessment. (PDF 20 kb) [file 12909_2019_1532_MOESM3_ESM.pdf]

	Dyrbye / 2010 / 1016	Kjeldstadli / 2006 / 48	Kötter / 2016 / 646	Michalec / 2013 / 89	Voltmer / 2012 / 840	Yusoff / 2013 / 39
**Representativeness of the exposed cohort**	no description of the derivation of the cohort	truly representative of the average *medical student* in the community	somewhat representative of the average medical student in the community	somewhat representative of the average *medical student* in the community	somewhat representative of the average medical student in the community	unclear
**Selection of the non exposed cohort**	drawn from the same community as the exposed cohort	drawn from the same community as the exposed cohort	drawn from the same community as the exposed cohort	drawn from the same community as the exposed cohort	drawn from the same community as the exposed cohort	drawn from the same community as the exposed cohort
**Ascertainment of exposure**	written self report	written self report	written self report	written self report	written self report	written self report
**Demonstration that outcome of interest was not present at start of study**	no	yes	yes	yes	no	no
**Comparability of cohorts on the basis of the design or analysis**	study controls for *gender* / multivariate analysis	study controls for *gender* / *age*	study controls for *gender* / multivariate analysis	study controls for *gender* / *skin colour, age*	study controls for gender / age	unclear
**Assessment of outcome**	self report	self report	self report	self report	self report	self report
**Was follow-up long enough for outcomes to occur (1 year)**	yes	yes	no	no	yes	yes
**Adequacy of follow up of cohorts**	follow up rate < 50% and no description of those lost	subjects lost to follow up unlikely to introduce bias (small number lost, > 50% follow up, or description provided of those lost)	subjects lost to follow up unlikely to introduce bias (small number lost, > 50% follow up, or description provided of those lost)	complete follow up - all subjects accounted for	follow up rate <50% and no description of those lost	no statement
