# Supplementary material for: What keeps medical students healthy and well? A systematic review of observational studies on protective factors for health and well-being during medical education
Source: BMC Med Educ. 2019 Apr 1;19:94. doi: 10.1186/s12909-019-1532-z (PMC6444449; doi:10.1186/s12909-019-1532-z)
Supplement: Supplementary file 4 — Table S2. Characteristics of the included studies. (PDF 44 kb) [file 12909_2019_1532_MOESM4_ESM.pdf]

	**Dyrbye / 2010 / 1016**	**Kjeldstadli / 2006 / 48**	**Kötter / 2016 / 646**	**Michalec / 2013 / 89**	**Voltmer / 2012 / 840**	**Yusoff / 2013 / 39**
**Study sites** (country / setting)	U.S. / 5 Medical Schools	Norway / 4 Medical Schools	Germany / 1 Medical School	U.S. / 1 Medical School	Germany / 1 Medical School	Malaysia / 1 Medical School
**Duration** (years)	1	6	0,75	0,75	5	1
**Outcome(s)** (instruments)	**Resilience** (no burnout [MBI] at either time point), **Recovery** (burnout at t0, no burnout at t1)	**Life satisfaction** (global one-item measure)	**General health** (one item), **Mental Health** (HADS)	**Mental health** (emotional well-being [EWB]/ personal well-being PWB]/ social well-being [SWB]), **MHC-SF**	**Anxiety**, **Depression** (HADS)	**Stress**, **Anxiety**, **Depression** (DASS-21)
**Independent variables** (instruments)	personal characteristics, major stressful life events, social support, learning climate, fatigue (ESS), stress (PSS)	Personality (BCI), Medical school stress (PMSS), Coping (WCCL)	Age, gender, personality (NEO-FFO), study-related behaviour and experience (AVEM), leisure time activities	Gender, age, race, year in the school, Medical School stressors (financial worries, lack of time / time management, academic pressures from self and others / fear of failure, amount of material to learn / academic demands, how you are evaluated, demands of social and intimate relationships, peer competition, powerlessness in system / anonymity in program, perceived mistreatment by faculty,	Work-related Behavior and Experience Patterns (AVEM), Perceived medical school stress, Short Form-12 Health Survey (SF-12), M1 Grade, demographic questions	Demographic profile, Emotional intelligence (USMEQ-i), Personality (USmaP-i)
**n** (t0)**, response rate**	1701 of 3080 (55%)	375 of 421 (89%)	359 of 376 (95%)	237 of 246 (96%)	112 of 182 (62%)	196 of 196 (100%)
**n** (longitudinal sample)**, response rate**	792 of 3080 (26%)	236 of 421 (56%)	308 of 376 (60%)	223 of 246 (91%)	42 of 182 (23%)	167 of 196 (85%)
**Statistical method(s)**	Logistic regression	Analysis of variance (ANOVA) and logistic regression analyses	Binary logistic regression analyses	Regression analyses, sensitivity analyses	Analyses of variance for repeated measures	Multiple linear regression test (stepwise method)
**Identified protective factors**	**Resilience:** non-white, increased satisfaction with the learning environment, greater agreement that student education is a priority for faculty members, lower fatigue, lower stress; **Recovery:** non-white, greater agreement that student education is a priority for faculty members, lower stress, working for income	low levels of stress in the form of one's perception of social and personal renunciations, low levels of wishful thinking as a way of coping	**General health:** age, striving for perfection, balance and mental stability, satisfaction with life, physical activity; **Mental health:** age, emotional distancing, experience of social support, neuroticism, relaxation techniques	**EWB:** T1 EWB, change in PWB, T1 SWB, change in SWB, overall negativ impact of stressors; **PWB:** T1 EWB, change in EWB, T1 PWB, T1 SWB, change in SWB; **SWB:** change in EWB, T1 PWB, change in PWB, T1 SWB	**Anxiety:** Physical health (SF-12), PMSS sum score, Risk pattern B; **Depression:** PMSS sum score, Risk pattern B	**Stress:** Neuroticism; **Anxiety:** Neuroticism, Emotional expression; **Depression:** Neuroticism
**Risk estimate** (with confidence interval / measure of variation) **for each protective factor**	OR (95% CI) **Resilience:** non-white 1.92 (1.23–2.99), increased satisfaction with the learning environment 1.52 (1.21–1.90), greater agreement that student education is a priority for faculty members 1.33 (1.02–1.74), lower fatigue 0.93 (0.89–0.98), lower stress 0.86 (0.83–0.88); **Recovery:** non-white 2.34 (1.18–4.64), greater agreement that student education is a priority for faculty members 1.59 (1.67–2.18), lower stress 0.86 (0.82–0.91), working for income 0.35 (0.13–0.95)	OR (95% CI) PMSS - social and personal renunciations 0.76 (0.68-0.85), WCCL - wishful thinking 0.93 (0.87-1.0)	OR (95% CI) **General health:** age 0.85 (0.74-0.98), striving for perfection 0.76 (0.66-0.88), balance and mental stability 1.20 (1.05-1.36), satisfaction with life 1.18 (1.01-1.38), physical activity 4.58 (2.09-10.05); **Mental health**: age 0.85 (0.74-0.99), emotional distancing 1.25 (1.03-1.50), experience of social support 0.73 (0.57-0.94), neuroticism 0.89 (0.84-0.95), relaxation techniques 0.26 (0.10-0.70)	*B* (no CI reported, p < .05) **EWB:** T1 EWB 0.40, change in PWB 0.14, T1 SWB 0.12, change in SWB 0.11, overall negativ impact of stressors -0.11; **PWB:** T1 EWB 0.51, change in EWB 0.53, T1 PWB 0.53, T1 SWB 0.29, change in SWB 0.51; **SWB:** change in EWB 0.38, T1 PWB 0.15, change in PWB 0.46, T1 SWB 0.58	OR (95% CI) **Anxiety:** Physical health (SF-12) -0.11 (-0.20--0.01), PMSS sum score 0.14 (0.07-0.22), Risk pattern B 3.98 (2.25-5.72); **Depression:** PMSS sum score 0.15 (0.09-0.21), Risk pattern B 2.02 (0.53-3.51)	*B* (95% CI) **Stress:** Neuroticism 0.55 (0.35-0.75); **Anxiety:** Neuroticism 0.54 (0.32-0.77), Emotional expression 3.69 (0.45-6.94); **Depression:** Neuroticism 0.46 (0.26-0.66)
**Self reported conclusion** of the study	"Social support, stress reduction, adequate rest and the removal of the additional burden of paid employment are likely to contribute to resiliency to burnout among students. Our study suggests that specific modifiable aspects of the learning climate are strongly related to variations in resiliency to burnout."	"Medical students who sustained high levels of life satisfaction perceived medical school as interfering less with their social and personal life, and made less use of passive, emotion focused coping, such as wishful thinking, than did their peers. Medical schools should encourage students to try to achieve a balance between schoolwork and their social and personal lives, and emphasise the importance of healthy coping strategies, for instance, by providing stress management courses."	"Before the beginning of courses, medical students rated their general and mental health mostly as good. Both parameters declined throughout the first year of medical education, and a more marked decline was shown in female students. In line with the conceptual framework applied, certain personality and temperament factors, such as balance and mental stability, as well as the ability to distance oneself from work, and physical activity as a proposed positive input to the medical student's coping reservoir, proved to be predictors of the maintenance of good general health. These findings may help in the development of health-promoting interventions in the future (e.g. the provision of time slots for physical activity)."	"Gains in SWB, however, may lend to positive benefits in other aspects of well-being."	None relating to the identified protective factors.	"This study found that neuroticism was the strongest associated factor of psychological health of medical students during the most stressful period."
**Self reported limitations** of the study	"Firstly, perceptions of social support and the learning climate were assessed only at baseline. It is possible that student perceptions of their social support varied between time-points. Secondly, employment, stress and fatigue were assessed only at the 1-year follow-up. Accordingly, our study was well designed to evaluate the role of fatigue and stress in perpetuating burnout, but was less able to evaluate how they contribute to the development of burnout in the first place. We are unable to determine whether employment, stress and fatigue are causally related to resiliency. Thirdly, although our response rate is typical of doctor and medical student surveys, our study is also potentially limited by response bias. We do not know how burnout influences response rate. Students with burnout may be more likely to complete surveys on student well-being as the topic is relevant to them, or, alternatively, they may be more apathetic and less likely to complete a survey. Fourthly, we assessed a limited number of social supports, learning climate characteristics and personal life events. Other unexplored factors in these domains may also relate to student resiliency."	"The response rates in the cross-sectional samples of our study were quite high, and although the response rate was somewhat lower in the longitudinal sample, it still consisted of more than half of all the students who entered medical school in Norway in 1993." "The data on the control groups were collected midway through the study. The comparisons made between the students in their first and last years are hence done with data of other students collected three years later and three years earlier, respectively." "Another limitation of the study is the employment of a single item as an outcome measure, which may reduce the reliability of the responses, but we consider its correlation with a validated subjective wellbeing scale to be satisfactory."	"Unfortunately, despite high response rates in both the t0 and t1 surveys (95% and 83%, respectively), 83 datasets could not be matched because they were identified by implausible or incorrect pseudonyms." "The slight over-representation of women in the longitudinal cohort is a well-known phenomenon and may have led to some selection bias." "As the study was intended to examine predictors of self-rated health for the freshman year of medical school, no objective data on health status were collected. No valid medical diagnoses can be made on the basis of the data collected. The predictive model could have been improved by including objective measures, such as blood chemistry results or bodily examination parameters." "The generalisability of the present results may be limited by the single-centre nature of the study."	"A particular limitation of this study is that only one medical school was sampled. Although it could be argued that all medical students, regardless of institution, share similar stressors, sampling from other schools would have provided a more representative sample of medical students. Also, this study looks at well-being scores spanning only 9–10 months. A more in-depth investigation of the potential changes in mental health among medical students would assess students more frequently to not only highlight when the potential change initiates, but also how scores fluctuate, especially concerning EWB."	"While this is one of the rare longitudinal studies across almost the whole course of medical education, the number of students who could be analyzed across all three measure points was small. One reason was the unreliable self-generated coding. Another reason may have been, that a number of students did not reach the fifth semester in time or left/changed university. The German version of the perceived medical school stress instrument was not available at earlier measure points. Future studies will have to show the development of medical school stress. It must also be noted that we report results from medical students from only one German university, and, consequently, these results may not be representative for all German medical schools or medical students."	First, this study involved a relatively small number of study subjects that might compromise the accuracy of the results obtained from the analysis. Second, this study was confined to a cohort of medical students of one medical school and in one country, limiting the generalisability of its results. Third, the sampling method was non-probability, which might lead to sampling bias that may compromise the results obtained. Fourth, several measurement constructs had Cronbach's alpha less than 0.7 that indicate a relatively low level of reliability. Last, psychological health measurement was collected through the face-to-face method, which was not fully anonymous and may lead to response bias.
**Other** limitations of the study	--	--	--	--	--	--
